# The Auxin Signaling Repressor IAA8 Promotes Seed Germination Through Down-Regulation of *ABI3* Transcription in *Arabidopsis*

**DOI:** 10.3389/fpls.2020.00111

**Published:** 2020-02-20

**Authors:** Shah Hussain, Sun Ho Kim, Sunghwa Bahk, Akhtar Ali, Xuan Canh Nguyen, Dae-Jin Yun, Woo Sik Chung

**Affiliations:** ^1^Division of Applied Life Science (BK21 Plus program), Plant Molecular Biology and Biotechnology Research Center, Gyeongsang National University, Jinju, South Korea; ^2^Department of Biomedical Science and Engineering, Konkuk University, Seoul, South Korea; ^3^Faculty of Biotechnology, Vietnam National University of Agriculture, Hanoi, Vietnam

**Keywords:** *ABI3*, *Arabidopsis*, auxin, IAA8, protein stability, seed germination

## Abstract

Seed germination is a complex biological process controlled by various regulators, including phytohormones. Among these, abscisic acid and gibberellic acid inhibit and promote seed germination, respectively. Many studies have addressed the biological roles of auxin in plant growth and development, but very few have considered its role in seed germination. Here, we identified a novel function of the auxin signaling repressor Aux/IAA8 during seed germination. The *IAA8* loss-of-function mutant *iaa8-1* exhibited delayed seed germination. The phenotype of *iaa8-1* was restored by ectopic expression of *IAA8*. Interestingly, IAA8 accumulated to high levels during seed germination, which was achieved not only by increased protein synthesis but also by the stabilization of IAA8 protein. We also showed that IAA8 down-regulates the transcription of *ABSCISIC ACID INSENSITIVE3* (*ABI3*), a negative regulator of seed germination. Our study, thus strongly suggest that the auxin signaling repressor IAA8 acts as a positive regulator of seed germination in *Arabidopsis thaliana*.

## Introduction

Seed maturation is the final stage of embryogenesis. The embryo becomes protected by a hard outer cover of dead tissue, the testa, underneath which the endosperm is deposited (Debeaujon et al., 2000). Upon germination, the testa ruptures, allowing the embryonic axis to protrude at the proper time to ensure propagation to the next generation (Piskurewicz et al., 2008). Germination occupies a critical position in the life cycle of seed plants, converting dormant seeds into active seedlings. This process is strictly regulated by genetic and environmental factors (Chen et al., 2008; Bassel et al., 2011; Liu et al., 2013). From an agronomic point of view, uniform germination is required for high crop yield. However, lack of dormancy may lead to pre-harvest sprouting (Gubler et al., 2005), resulting in decreased seed longevity (Rajjou and Debeaujon, 2008). Therefore, it is important to maintain seeds in a dormant condition until suitable timing for germination is established (Shu et al., 2016).

Releasing dormancy is a prerequisite to germination that can be induced by various external and internal stimuli (Arc et al., 2012). Germination is induced most commonly through the imbibition of water at a species-specific temperature. Imbibition of dry seeds activates a series of events (Bewley, 1997) including oxidation, degradation, and mobilization of accumulated reserve components (Penfield et al., 2005a). Reactive oxygen species (ROS) also accumulate in seeds to a level that positively regulates seed germination (Leymarie et al., 2012). ROS are proposed to up-regulate abscisic acid (ABA) catabolism and promote gibberellic acid (GA) biosynthesis, thereby maintaining a dynamic balance between ABA and GA during seed germination (Liu et al., 2010).

Various endogenous regulators including phytohormones and associated proteins control the transition from dormant to active, germinating seeds (Shu et al., 2016; Nee et al., 2017). Among phytohormones, ABA is considered to maintain seed dormancy and inhibit seed germination (Hubbard et al., 2010). Meanwhile, GA, brassinosteroids, ethylene, and cytokinin promote seed germination (Steber and McCourt, 2001; Wang et al., 2011). Several ABA signaling genes such as *ABSCISIC ACID INSENSITIVE3* (*ABI3*), *ABI4*, and *ABI5* was identified as negative regulators of seed germination. Among these, *ABI3* acts as a major downstream components of ABA signaling (Giraudat et al., 1992; Bentsink and Koornneef, 2008). The transcript levels of *ABI3* are high in dormant seeds but low after germination (Liu et al., 2013). The antagonistic roles of ABA and GA in dormancy and germination have been studied extensively (Shu et al., 2016). However, the relationship between ABA and auxin in seed germination remains unresolved.

Auxin is involved in almost every aspect of plant growth and development (Zhao, 2010); however, its role in seed germination is still unknown. Rapid turnover of auxin/indole-3-acetic acid (Aux/IAA) repressor proteins is required (Overvoorde et al., 2005) to trigger auxin-mediated transcriptional activation (Tiwari et al., 2003). These short-lived transcriptional repressors are mainly targeted for degradation by polyubiquitination (Kepinski and Leyser, 2005; Overvoorde et al., 2005; Gilkerson et al., 2015). Recent reports have suggested that auxin inhibits seed germination in an ABA dependent manner. For instance, seeds of auxin over-producing transgenic plants (*iaaM-OX*) display arrested germination (Liu et al., 2013). By contrast, mutants with reduced levels of auxin (*yuc1/yuc6*) showed enhanced seed germination rate (Liu et al., 2013). Similarly, auxin signaling mutants with impaired ability to degrade Aux/IAA repressor proteins show enhanced germination (Kepinski and Leyser, 2005; Liu et al., 2013). Gain-of-function mutants of Aux/IAA repressors, such as IAA7/AXR2 and IAA17/AXR3, also display enhanced germination rate (Liu et al., 2013). The underlying genetic and biochemical evidence suggests that *ABI3* is the downstream regulatory component of auxin-mediated seed dormancy (Belin et al., 2009; Liu et al., 2013). These molecular observations imply that inhibition of auxin signaling *via* Aux/IAA might be responsible for promoting seed germination.

Although the gain-of-function mutation of IAA8 negatively regulates flower development (Wang et al., 2013), the loss-of-function mutant show no visible developmental phenotype (Overvoorde et al., 2005). To decipher the molecular mechanism explaining how auxin signaling regulates seed germination, we characterized the biological role of IAA8 during seed germination. We provide evidence that IAA8 protein accumulates during seed germination, promoting germination through the inhibition of *ABI3* transcription.

## Materials and Methods

### Plant Material and Growth Conditions

*Arabidopsis thaliana* ecotype Columbia (Col-0) was used in all experiments. T-DNA insertion mutants *iaa8-1* (CS25210) and *iaa8-2* (SALK_202296) were obtained from SALK. T-DNA insertion was confirmed by genotyping PCR using *IAA8* gene-specific and T-DNA border primers (listed in **Supplementary Table 1**). The transcript was confirmed by semi-quantitative RT-PCR using *IAA8* gene specific forward and reverse primers (**Supplementary Table 1**).

Seeds were surface sterilized and then stratified at 4°C for 4 days in the dark. All seeds were germinated on plates containing half-strength Murashige and Skoog (½ MS) medium supplemented with 2% sucrose and 0.25% Phytagel. Plates were then transferred to a growth chamber at 22 ± 2°C under long day conditions (16-h-light/8-h-dark photoperiod) with 100 E m^−2^ s^−1^ light intensity.

### Generation of Transgenic Plants Overexpressing *IAA8*

The *Cauliflower mosaic virus* (*CaMV*) *35S::3xflag-IAA8* construct in binary vector pCAMBIA 1300 was introduced into *Agrobacterium tumefaciens* strain GV3101 and used for transformation of *iaa8-1* mutant plants by floral dipping. Transformed lines were selected on ½ MS medium containing hygromycin (40 g/mL). Three independent homozygous lines overexpressing *IAA8* were selected from the T_3_ generation and used for all experiments.

### Seed Germination Assay

Seeds were carefully harvested after siliques were fully mature. The germination assay was performed according to the method of Nguyen et al. (2012). After surface sterilization, seeds of all genotypes were stratified at 4°C for 4 days in the dark and allowed to germinate on ½ MS medium or ½ MS supplemented with 5 µM NAA or 1 µM ABA alone or together at 22 ± 2°C in a growth chamber under a 16-h-light/8-h-dark cycle. Seed germination based on radicle protrusion was quantified from day 0 until day 5. Seeds were considered germinated after radicle protrusion at the indicated time. Statistical analysis was performed, and data are presented as percentage germination rate from three independent experiments with three biological replicates.

### Protein Extraction and Immunoblot Analysis

Immunoblot analysis was performed according to the method of Kim et al. (2017). Seedlings were treated with or without MG132, cycloheximide (CHX), or H_2_O_2_. Tissues were ground in liquid nitrogen to fine powder, and total proteins were extracted using extraction buffer containing 50 mM HEPES, pH 7.5, 5 mM EDTA, 5 mM EGTA, 2 mM DTT, 25 mM NaF, 1 mM Na_3_VO_4_, 50 mM β-glycerophosphate, 20% glycerol (v/v), 2 mM PMSF, 1% Triton X-100 (v/v), and protease inhibitor cocktail (Roche diagnostics, Germany). Following two rounds of centrifugation at 12,000 × *g* for 15 min, supernatants were transferred to pre-chilled micro centrifuge tubes. Protein concentration was determined using a protein assay kit (Bio-Rad Laboratories, USA) with bovine serum albumin (BSA) as a standard. For immunoblot analysis, 80 µg of total protein from each sample was separated by 10% Sodium Dodecyl Sulfate Polyacrylamide Gel Electrophoresis (SDS-PAGE) and transferred to polyvinylidene fluoride (PVDF) membranes (Bio-Rad Laboratories, USA). Proteins were probed using mouse anti-flag (1:5000; Sigma, USA) as primary antibody and horseradish peroxidase (HRP) conjugated anti-mouse as secondary antibody (1:5000) and visualized using an ECL kit (Bio-Rad Laboratories, USA).

### RNA Extraction and Gene Expression Analysis by Reverse-Transcription Quantitative PCR (RT-qPCR) and Semi-Quantitative RT-PCR

Total RNA was extracted from seeds by the LiCl/phenol method according to the protocol of Nguyen et al. (2012). RNA (2 µg) was reverse transcribed using SuperScript II RNase-Reverse Transcriptase (Invitrogen, USA). RT-qPCR was performed according to the method of Kim et al. (2017) with some modification. RT product (1 µL) was mixed with gene specific primers (10 pmol) in 10 µL reaction volume. SYBR Green PCR Master Mix kit (Bio-Rad SYBR Green Supermix) was added to the mixture and incubated in a CFX384 real-time PCR detection system (Bio-Rad Laboratories, USA). Gene expression was quantified during the logarithmic phase using expression of the housekeeping gene *Tubulin2* as an internal control. Semi-quantitative RT-PCR was carried out as described by Nguyen et al. (2012). Primers used for PCR are listed in **Supplementary Table 2**.

### Chromatin Immunoprecipitation Assay

Ten-day-old *iaa8-1* and *iaa8-1*/*IAA8 OX* seedlings were treated with cold (4°C) and H_2_O_2_ for 12 h. Chromatin immunoprecipitation (ChIP) was carried out as described (Gendrel et al., 2002) using mouse polyclonal anti-flag antibody (1:3000; Sigma, USA). PCR amplification was performed quantitatively using the CFX384 Real-Time System (Bio-Rad, USA). The immunoprecipitation was replicated three times. The ChIP primers are listed in **Supplementary Table S2**.

## Results

### IAA8 Is Involved in Seed Germination

IAA8 negatively regulates flower development (Wang et al., 2013). To investigate the biological function of IAA8, we obtained the T-DNA insertion mutants *iaa8-1* (CS25210) and *iaa8-2* (SALK_202296) from SALK (**Supplementary Figure 1A**) and confirmed the T-DNA insertion by diagnostic PCR and semi-quantitative RT-PCR assays (**Supplementary Figure 1B**). The full length transcript of *IAA8* was disrupted in the *iaa8-1* line, whereas the transcript in *iaa8-2* was similar to the Col-0 plants, suggesting that only *iaa8-1* is a loss-of-function mutant (**Supplementary Figure 1C**).

**Figure 1:**
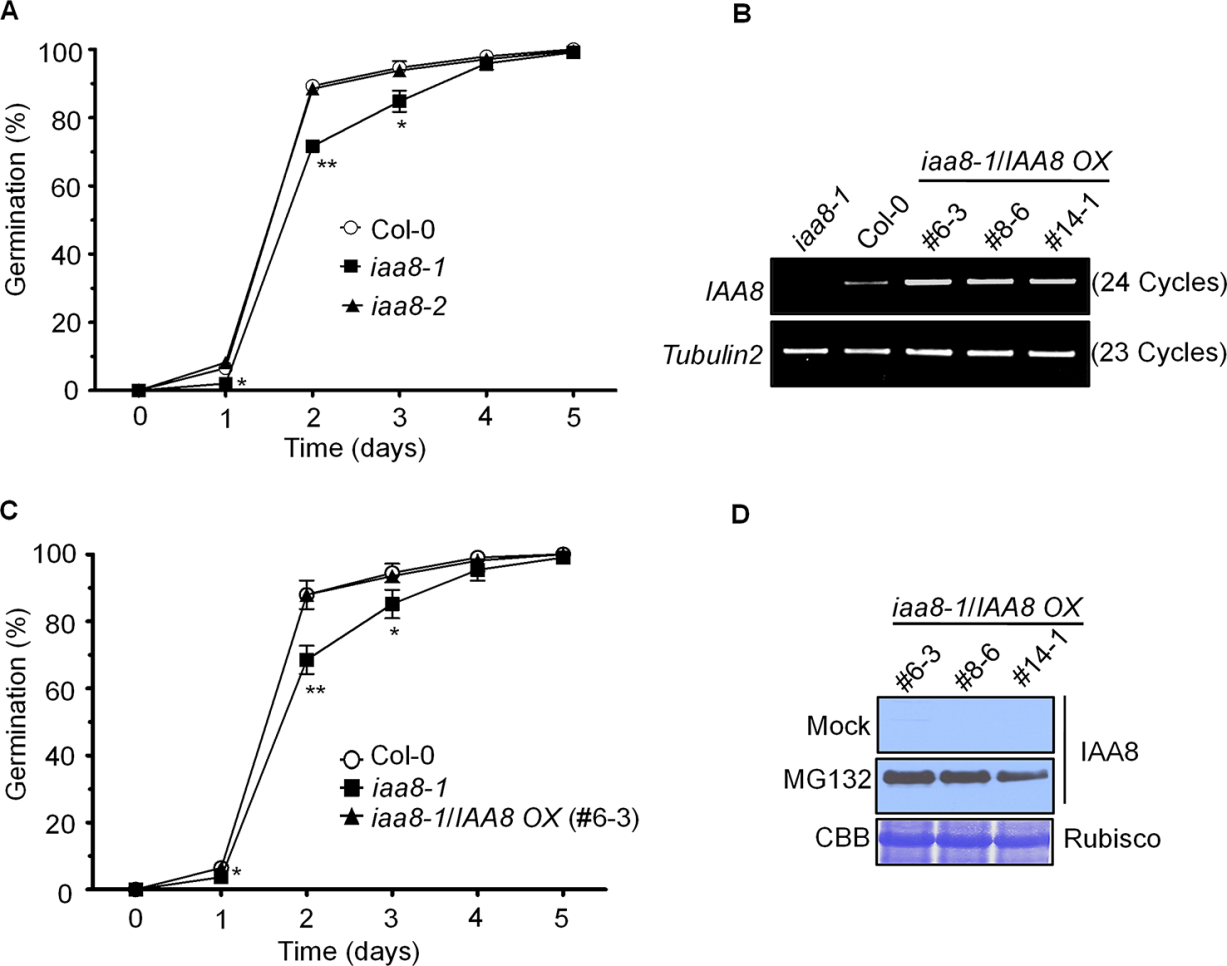
IAA8 positively regulates seed germination. **(A)** Seeds of Col-0, *iaa8-1*, and *iaa8-2* mutants were germinated on ½ MS medium after stratification for 4 days in the dark at 4°C in a long-day chamber. Radicle protrusion was quantified at the indicated times (days). Data are presented as mean values of three independent experiments of three biological replicates. Error bars represent SE. Significant difference was determined by Student's t-test (*P < 0.05 and **P < 0.01). **(B)** Construction of transgenic lines overexpressing *IAA8* in the *iaa8-1* background. Abundance of *IAA8* transcript in Col-0 and transgenic lines expressing *p35S::3xflag-IAA8* was determined by semi-quantitative RT-PCR. Total RNA was extracted from 10-day-old seedlings, and semi-quantitative RT-PCR analysis was performed using gene-specific primers; *Tubulin2* was used as an internal control. **(C)** *IAA8* rescues the germination phenotype of *iaa8-1*. Seeds of Col-0, *iaa8-1*, and an *iaa8-1/IAA8 OX* (#6-3) complemented line were germinated on ½ MS medium in a long-day chamber. Radicle protrusion was quantified at the indicated times (days). Data are presented as mean values. Error bars represent SE. Significant difference was determined by Student's t-test (*P < 0.05 and **P < 0.01). **(D)** Immunoblot analysis of protein extracts prepared from samples used in **(B)** to confirm the expression of flag-tagged IAA8 protein in the absence (mock) or presence of 10 µM MG132. Proteins were extracted and probed with anti-flag antibody. Rubisco was stained with Coomassie brilliant blue (CBB) and used as a loading control.

To identify the physiological function of *IAA8*, we intensively screened the *iaa8-1* phenotype. In a time course experiment, seeds of the selected genotypes were germinated on ½ MS medium for 5 days under long day conditions. Germination rate based on radicle protrusion was lower in *iaa8-1* mutant seeds than in Col-0 and *iaa8-2* seeds (**Figure 1A**), indicating that *IAA8* positively regulates seed germination.

To further confirm the *iaa8-1* mutant phenotype, we generated complementation plants expressing *p35S::3xflag-IAA8* in the *iaa8-1* background (*iaa8-1*/*IAA8 OX*). Three independent homozygous lines were selected from the T_3_ generation. To verify ectopic expression, semi-quantitative RT-PCR and immunoblot analyses were performed (**Figures 1B, D**). Aux/IAA repressor proteins are rapidly degraded by the auxin-mediated 26S proteasomal pathway, thereby regulating auxin-dependent transcriptional regulation (Tiwari et al., 2003; Park et al., 2011). We assumed that IAA8 might also be degraded by the 26S proteasomal pathway, similar to other Aux/IAA proteins (Gilkerson et al., 2015). To test this hypothesis, 10-day-old seedlings of *iaa8-1/IAA8 OX* plants from three independent lines were treated with or without 10 µM MG132 (a proteasome inhibitor) for 3 h. Proteins were extracted and immunoblot analysis was performed. As expected, IAA8 protein was almost undetectable under mock conditions (control); however, high levels of IAA8 protein accumulated in the presence of MG132 (**Figure 1D**), indicating that IAA8 is degraded by the 26S proteasomal pathway.

We next tested seed germination phenotypes using Col-0, *iaa8-1*, and three independent *iaa8-1*/*IAA8 OX* complemented lines. Seeds of the selected genotypes were allowed to germinate on ½ MS medium under long day conditions. The germination rate of *iaa8-1*/*IAA8 OX* complemented seeds was almost the same as that of Col-0 (**Figure 1C** and **Supplementary Figure 2**), indicating that the lower germination in the *iaa8-1* mutant line resulted from loss-of-function of *IAA8*. Three independent complemented lines showed similar expression patterns and seed germination phenotype. Therefore, we used the *iaa8-1*/*IAA8 OX* (#6-3) for further experiments, and hereafter referred to as *iaa8-1*/*IAA8 OX*.

Previous study suggests that auxin inhibits seed germination through ABA-dependent manner (Liu et al., 2013). Therefore, we tested the effect of auxin on germination by using Col-0, *iaa8-1*, and *iaa8-1*/*IAA8 OX* seeds in the presence and absence of ABA (**Supplementary Figure 3**). Seeds of the selected genotypes were allowed to germinate on ½ MS or ½ MS supplemented with 5 µM NAA and 1 µM ABA alone or together. In our experimental condition, exogenous NAA alone did not inhibit seed germination in Col-0 and *iaa8-1/IAA8 OX* compared to *iaa8-1* mutant (**Supplementary Figures 3A, B**). These results suggest that even upon NAA treatment, small amount of IAA8 protein can be existed in Col-0 and *iaa8-1/IAA8 OX*, which inhibits *ABI3* expression to promote seed germination compared to *iaa8-1* mutant seeds. However, co-treatment of NAA and ABA synergistically inhibited seed germination in Col-0 and *iaa8-1/IAA8 OX* seeds similar to those of *iaa8-1* (**Supplementary Figures 3C, D**), indicating that *ABI3*, *ABI4*, and *ABI5* transcription by ABA may cause inhibitory effect on germination, which cannot be rescued by small amount of IAA8 proteins.

### IAA8 Protein Accumulates During Seed Germination

**Figure 2:**
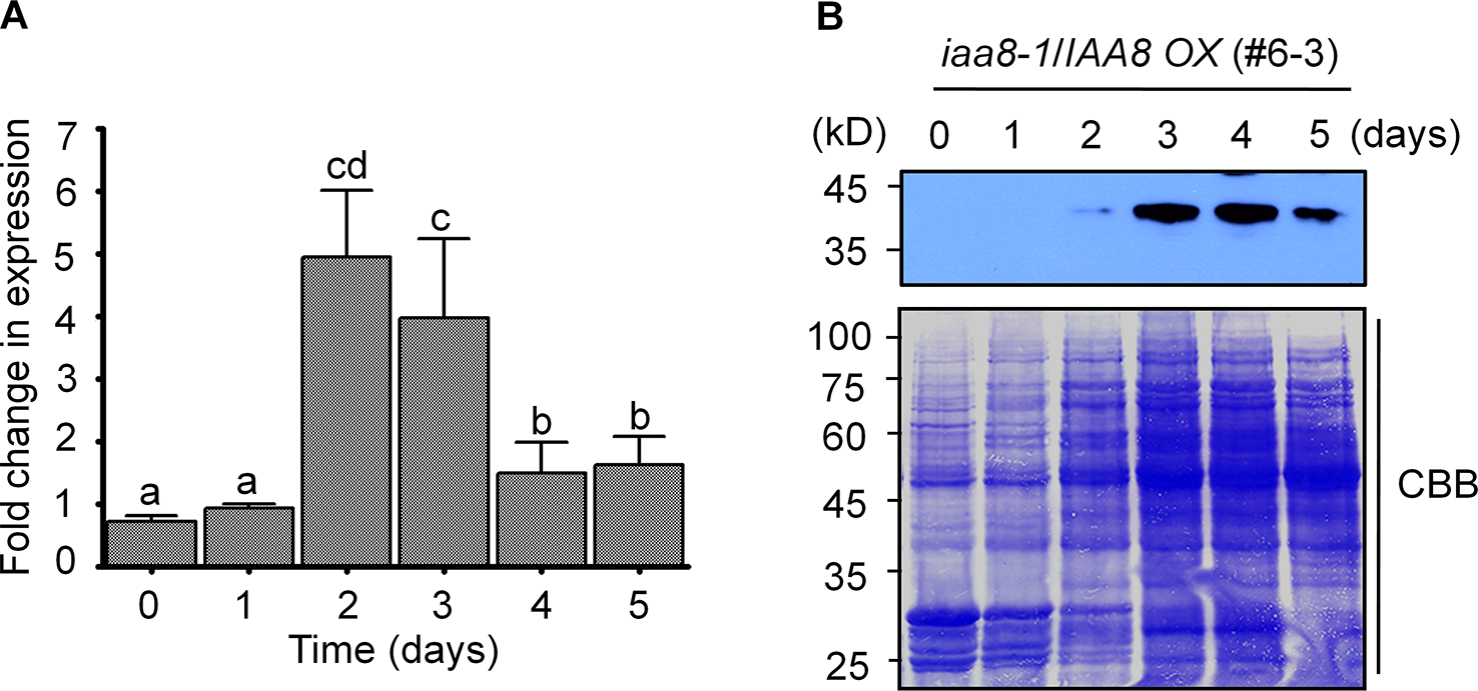
*IAA8* transcript levels and protein abundance during seed germination. **(A)** *IAA8* transcript level in germinating seeds of Col-0 plants. Seeds of Col-0 plants were germinated on ½ MS medium for the indicated time periods (days). Total RNA was extracted, and RT-qPCR analysis was performed using gene-specific primers; *Tubulin2* was used as an internal control. Data are presented as mean values. Error bars represent SE of three biological replicates. Different letters indicate statistically significant differences (P < 0.05). **(B)** IAA8 protein accumulation during seed germination. Seeds of *iaa8-1*/*IAA8 OX* (#6-3) plants were stratified for 4 days in the dark at 4°C and then allowed to germinate on ½ MS medium. Proteins were extracted at the indicated time points (days), and immunoblot analysis was performed using 80 µg of total protein in each well. IAA8 protein was detected using anti-flag antibody. Membranes were stained using Coomassie brilliant blue (CBB). A representative image of three independent experiments is shown.

To further investigate the specific role of *IAA8* in seed germination, we searched the public *Arabidopsis* microarray database (http://bar.utoronto.ca/eplant/) and found that the *IAA8* transcript is induced by seed imbibition. We therefore proposed that *IAA8* transcription might be induced during seed germination. To determine the expression pattern of *IAA8* during seed germination, we performed RT-qPCR using freshly harvested Col-0 seeds. The *IAA8* transcript level peaked in germinating seeds at day 2 (**Figure 2A**), indicating that *IAA8* transcription is induced during seed germination.

Because *IAA8* transcription was induced during seed germination, we assumed that the IAA8 protein might also accumulate following initiation of germination signals. We therefore germinated seeds of *iaa8-1*/*IAA8 OX* plants and performed immunoblot analysis. As expected, IAA8 protein accumulated to high levels during seed germination. Interestingly, IAA8 protein was detectable at day 2 and accumulated rapidly until day 5 (**Figure 2B**). By day 10, however, IAA8 protein was almost undetectable (**Figure 1D**), suggesting that IAA8 proteins accumulate only during germination.

### IAA8 Protein Stability Is Maintained by Cold and ROS Signals

**Figure 3:**
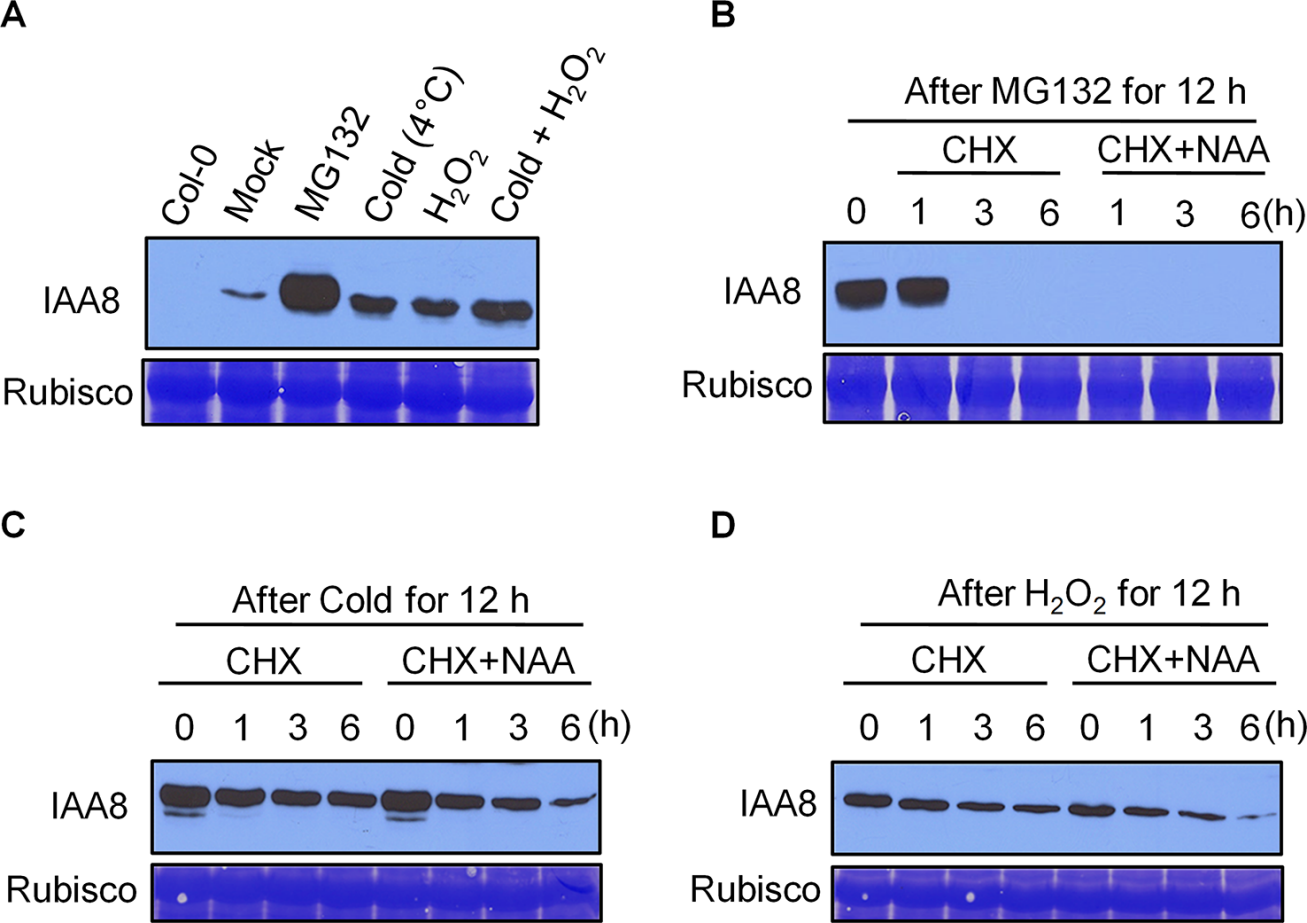
IAA8 protein is stabilized by cold and H_2_O_2_. **(A)** Stabilization of IAA8 in the presence of MG132, cold, and H_2_O_2_. Ten-day-old *iaa8-1*/*IAA8 OX* (#6-3) seedlings were treated with 10 µM MG132, cold (4°C), 5 mM H_2_O_2_, or cold and H_2_O_2_ together for 12 h. **(B–D)** *De novo* protein synthesis and auxin-mediated turnover of stabilized IAA8 in presence of MG132, cold, or H_2_O_2_. Ten-day-old *iaa8-1*/*IAA8 OX* (#6-3) seedlings were treated with 10 µM MG132. **(B)** cold (4°C). **(C)** or 5 mM H_2_O_2_ **(D)** for 12 h and then incubated with 500 µM CHX with or without 20 µM NAA for the indicated time periods (h). Total proteins were extracted, and immunoblot analysis was performed using anti-flag antibody. Col-0 seedlings were used as a negative control. Rubisco was stained using Coomassie brilliant blue (CBB) and used as a loading control.

Cold treatment is considered one of the most efficient stimuli of seed dormancy release (Penfield et al., 2005b). Germinating seeds produce ROS upon imbibition, which promotes radicle protrusion by rupturing the testa (Leymarie et al., 2012). Cold and imbibition therefore work in parallel during seed germination. As IAA8 protein accumulated to high levels during seed germination after cold imbibition (**Figure 2B**), we investigated whether cold and ROS affect IAA8 protein level. Seedlings of *iaa8-1*/*IAA8 OX* plants were exposed to cold (4°C) and 5 mM H_2_O_2_ alone or together for 12 h. Total proteins were then extracted and immunoblot analysis was performed (**Figure 3**). Under mock conditions, IAA8 protein was detected at low levels, indicating that *iaa8-1*/*IAA8 OX* plants were under mild stress during experimental handling. However, treatment with cold or H_2_O_2_ induced a rapid accumulation of IAA8 (**Figure 3A**). Interestingly, more IAA8 protein accumulated when *iaa8-1*/*IAA8 OX* seedlings were incubated under cold and H_2_O_2_ together (**Figure 3A**). These results demonstrate that cold and ROS act together to promote IAA8 protein accumulation during germination.

We next asked whether accumulation of IAA8 protein under cold and H_2_O_2_ conditions was caused by *de novo* protein synthesis or increased protein stability. We exposed *iaa8-1*/*IAA8 OX* seedlings to MG132, cold or H_2_O_2_ for 12 h, and then incubated them in medium containing 500 µM cycloheximide (CHX; an inhibitor of translation) with or without 20 µM 1-naphthaleneacetic acid (NAA) for 1, 3, and 6 h. As expected, IAA8 protein accumulated rapidly in the presence of MG132; however, preventing *de novo* protein synthesis by treatment with CHX caused significant depletion of IAA8 (**Figure 3B**). Next, we treated *iaa8-1*/*IAA8 OX* seedlings with cold or H_2_O_2_, followed by incubation with CHX. Surprisingly, preventing *de novo* protein synthesis by treatment with CHX did not result in complete loss of IAA8 protein in cold and H_2_O_2_ treated seedlings (**Figures 3C, D**), indicating that the increased abundance of IAA8 protein caused by cold and H_2_O_2_ is independent of *de novo* protein synthesis. Furthermore, treatment with exogenous NAA potentiated the effect of CHX, leading to faster turnover of IAA8 in MG132-treated seedlings (**Figure 3B**). Surprisingly, however, IAA8 protein was still detectible after 6 h of NAA treatment in both cold- and H_2_O_2_-treated seedlings (**Figures 3C, D**). Together, these results suggest that cold and H_2_O_2_ delay auxin-mediated degradation of IAA8 protein.

### IAA8 Down-Regulates *ABI3* Expression During Seed Germination

**Figure 4:**
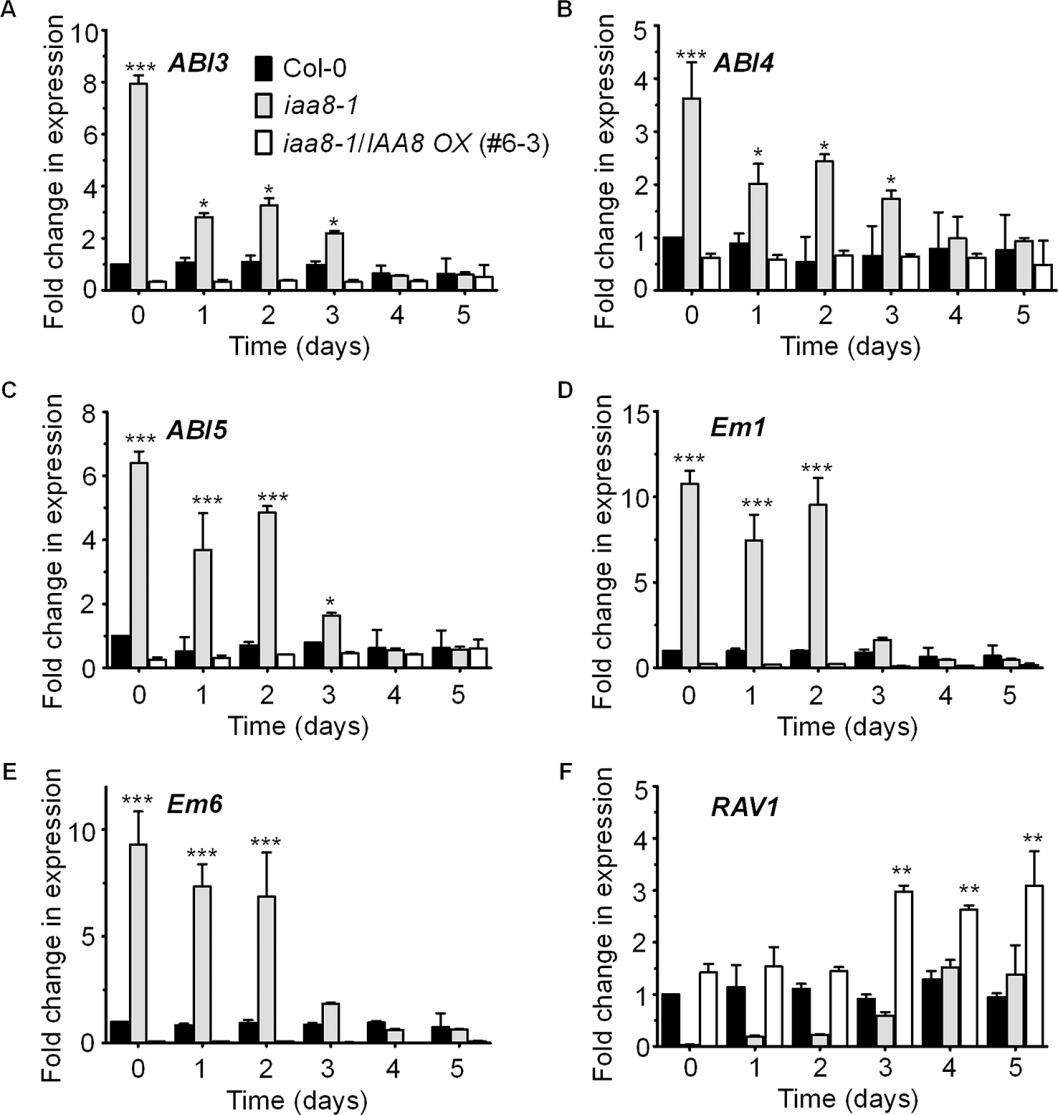
IAA8 down-regulates ABA responsive genes transcription. Transcript abundance of *ABI3* **(A)**, *ABI4* **(B)**, *ABI5* **(C)**, *Em1* **(D)**, *Em6* **(E)**, and *RAV1* **(F)** in germinating seeds of Col-0, *iaa8-1* mutant, and *iaa8-1/IAA8 OX* (#6-3) plants. Seeds of the selected genotypes were germinated on ½ MS medium. Total RNA was extracted and RT-qPCR analysis was performed using gene-specific primers; *Tubulin2* was used as an internal control. Error bars represent SE in three biological replicates. Significant difference was determined by Student’s t-test (*P < 0.05, **P < 0.01 and ***P < 0.001).

*ABI3* functions in ABA-mediated seed dormancy and inhibition of seed germination. *ABI3* transcript levels are high in dormant seeds and decrease rapidly after germination (Liu et al., 2013). Likewise, ABI3 protein levels decrease in germinating seeds in a light dependent manner (Lopez-Molina et al., 2002). We therefore proposed that IAA8 accumulation might regulate *ABI3* expression during or after seed germination. To test this hypothesis, we germinated seeds of Col-0, *iaa8-1*, and *iaa8-1*/*IAA8 OX* under long day conditions and collected samples from day 0 to day 5. Total RNA was then extracted, and RT-qPCR analysis was performed. Interestingly, the transcript levels of *ABI3* were comparatively high in non-germinating Col-0 seeds but declined during germination. The *ABI3* transcript levels were very high in *iaa8-1* seeds compared to those in Col-0 seeds (**Figure 4A**). Furthermore, the transcript levels of other ABA-responsive genes such as *ABI4*, *ABI5*, *Em1*, *Em6*, and *RAV1* were also analyzed. The results showed that transcript levels of *ABI4*, *ABI5*, *Em1*, and *Em6* were significantly elevated in *iaa8-1* (**Figures 4B–E**). In contrast, the transcript level of *RAV1* was very low in *iaa8-1* compared to Col-0 plants (**Figure 4F**). Consistent with the germination results (**Figures 1A, C**, **Supplementary Figure 2**), *IAA8* complementation down-regulated *ABI3*, *ABI4*, *ABI5*, *Em1*, and *Em6* expression to levels almost comparable to those in Col-0 (**Figure 4**), indicating that IAA8 negatively regulates *ABI3*, *ABI4*, *ABI5*, *Em1*, and *Em6* expression during seed germination.

**Figure 5:**
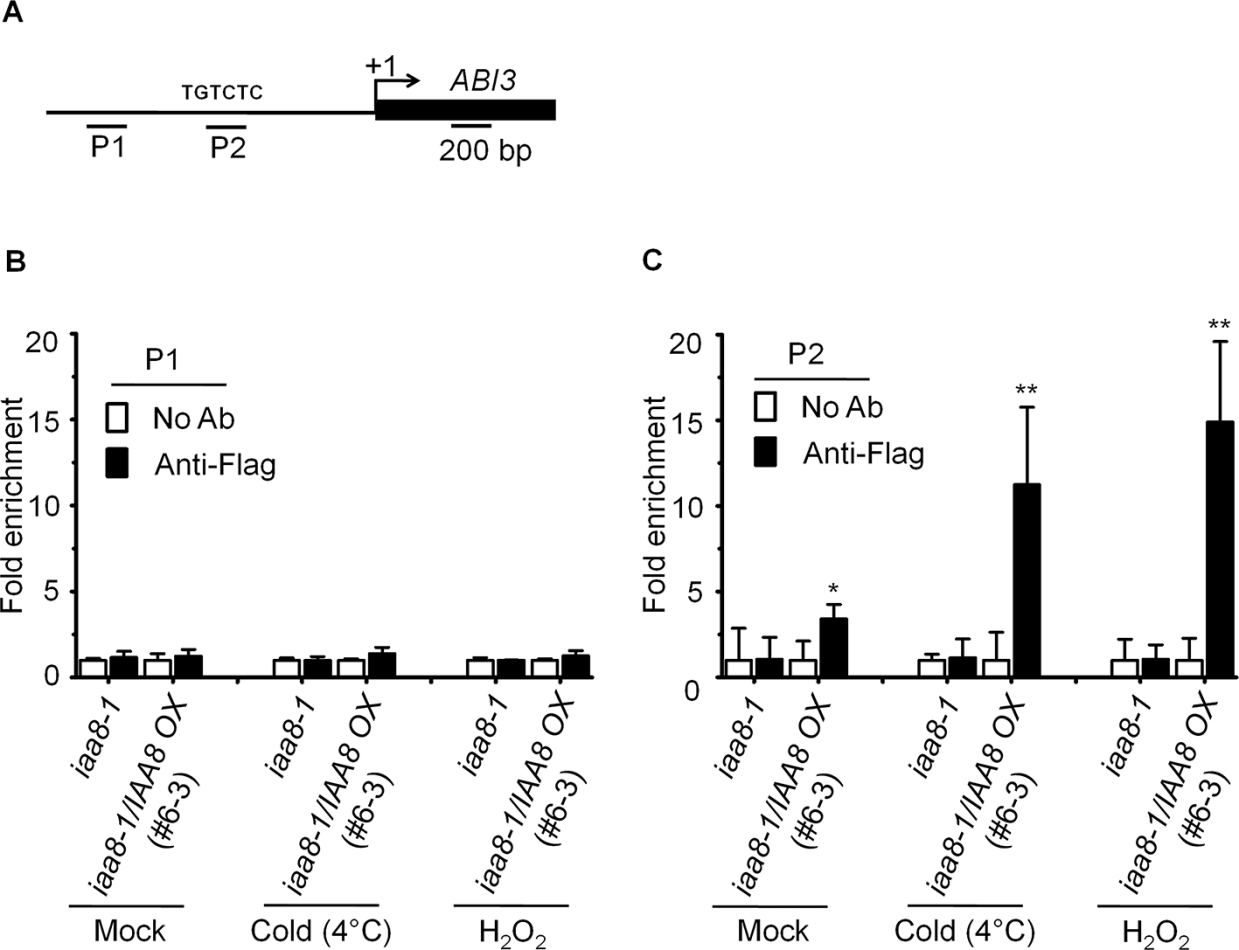
IAA8 associate to *ABI3* gene promoter. **(A)** Structure of the *ABI3* promoter and amplicon regions (P1 and P2). The arrow indicates the transcription start site. The black line and black rectangle indicate 1.5-kb promoter and CDS region, respectively. TGTCTC sequence is auxin responsive element (AuxRE). **(B–C)** Germination signals induce binding of IAA8 to *ABI3* promoter at AuxRE region. Chromatins from *iaa8-1* and *iaa8-1*/*IAA8 OX* (#6-3) plants were treated with cold (4°C) and H_2_O_2_ for 12 h. Flag-tagged IAA8 chromatin complex were immunoprecipitated with anti-Flag antibody. A control reaction was processed in parallel without antibody. ChIP-DNA was applied to RT-qPCR using primers specifically targeting to *ABI3* promoter regions, **(B)** (P1) and **(C)** (P2). The ChIP results are presented as fold-enrichment of nontarget DNA. Error bars indicate SE (*n* = 3). Significant difference was determined by Student's t-test (*P < 0.05 and **P < 0.01). The experiments were repeated three times with similar results.

Aux/IAA proteins are transcriptional repressors that regulate the expression of auxin responsive genes by inactivating ARFs activity (Tiwari et al., 2003). Recent studies suggest that *ABI3* functions as a major downstream regulatory component of auxin-mediated seed dormancy (Belin et al., 2009; Liu et al., 2013). We therefore asked how IAA8 might affect the expression of *ABI3*. For this purpose, chromatin immunoprecipitation (ChIP) assay was performed to confirm the binding ability of IAA8 with *ABI3* promoter using anti-flag antibody. We treated the *iaa8-1* and *iaa8-1*/*IAA8 OX* plants with mock, cold and H_2_O_2_ for 12 h, and then ChIP-qPCR analysis was performed. The results showed that IAA8 associates to AuxRE motif on *ABI3* promoter under mock condition in *iaa8-1*/*IAA8 OX* plants (**Figure 5**). Interestingly, the *ABI3* promoter fragment were highly enriched in *iaa8-1*/*IAA8 OX* plants in response to cold and H_2_O_2_ signals compared to ChIP samples in mock and without anti-flag treated plants (**Figure 5B**), suggesting that binding of IAA8 to *ABI3* promoter presumably through ARFs could down-regulate *ABI3* transcription during seed germination.

## Discussion

Seed germination is mainly controlled by two hormones (Leymarie et al., 2012): ABA inhibits germination, while GA promotes it. Catabolism of ABA and biosynthesis of GA are up-regulated by imbibition to release seeds from dormancy (Liu et al., 2010; Leymarie et al., 2012; Wang et al., 2018). Other hormones, including brassinosteroids, ethylene, and cytokinin, are also known to contribute to seed germination (Steber and McCourt, 2001; Wang et al., 2011; Corbineau et al., 2014). Auxin is generally considered to negatively regulate seed germination in an ABA-dependent manner (Liu et al., 2013). However, the biological role of auxin in seed germination has not been fully elucidated. In this study, we uncovered a novel physiological function of IAA8 in seed germination.

### IAA8 Is a Positive Regulator of Seed Germination

The biological functions of most *Aux/IAA* genes have been identified using gain-of-function mutants, with *axr5-1* (*iaa1*), *shy2* (*iaa3*), *iaa6* (*shy1*), *axr2-1*(*iaa7*), *iaa8*, *iaa12* (*bdl*), *iaa14* (*slr*), *iaa16*, *axr3-1*(*iaa17*), *iaa18*, *iaa19* (*msg2*), and *iaa28* reported to display auxin related phenotypes (Timpte et al., 1994; Kim et al., 1996; Leyser et al., 1996; Rouse et al., 1998; Hamann et al., 1999; Nagpal et al., 2000; Rogg et al., 2001; Fukaki et al., 2002; Tian et al., 2003; Tatematsu et al., 2004; Yang et al., 2004; Uehara et al., 2008; Rinaldi et al., 2012; Wang et al., 2013). Among these, the *IAA8* gain-of-function mutant negatively regulates flower development (Wang et al., 2013); however, the *IAA8* loss-of-function mutant shows no developmental phenotype in aerial plants parts (Overvoorde et al., 2005). In this study, we found that the *IAA8* loss-of-function mutant (*iaa8-1*) showed delayed seed germination compared to the wild type (**Figures 1A, C**). This result correlates well with previous observations. First, external auxin application negatively regulates seed germination (Liu et al., 2013). Second, the gain-of-function mutants *axr2-1* (IAA7) and *axr3-1* (IAA17) show enhanced seed germination and exhibit stronger ABA insensitivity than the wild type (Belin et al., 2009; Liu et al., 2013). Third, *tir1, tir1afb2, tir1/afb3*, and *tir1afb1afb2afb3* mutants with impaired ability to degrade Aux/IAA proteins show enhanced seed germination (Liu et al., 2013). These observations suggest that Aux/IAA proteins act as positive regulators of seed germination. The biological roles of other Aux/IAA proteins during seed germination should be investigated to better understand which and how many Aux/IAA proteins are involved.

### IAA8 Accumulation Is Caused by *De Novo* Synthesis and Increased Stability During Germination

The amount of different proteins in cells is controlled by translation of mRNA, which is dependent on gene expression. In addition, protein accumulation can also be achieved by increasing protein stability in response to external or internal signals (Finch-Savage et al., 2007; Carrera et al., 2008; Holdsworth et al., 2008). We found that IAA8 protein rapidly accumulated to high levels during seed germination in response to germination signals (**Figure 2B**). The increase in IAA8 protein was achieved not only by *de novo* protein synthesis but also by protein stabilization (**Figure 3**). Similarly, the expression of many germination related genes is highly up-regulated in response to germination-inducing signals (Wang et al., 2018). Several *Aux/IAA* genes, such as *IAA1*, *IAA2*, *IAA3*, *IAA16*, *IAA20*, *IAA26*, *IAA28*, and *IAA29*, are highly expressed during germination (Fujii et al., 2000; Winter et al., 2007; Carranco et al., 2010). Furthermore, transcription of *IAA30*, *IAA11*, and *IAA19* is enhanced under stress conditions in germinating seeds (Park et al., 2011), suggesting that many *Aux/IAA* genes may play important roles during seed germination in a stress-dependent or -independent manner.

This study showed that IAA8 protein is stabilized by cold and ROS signals during seed germination (**Figure 3**). Similarly, IAA7 and IAA17 are highly stabilized by salt and salicylic acid (Wang et al., 2007; Liu et al., 2015). Thus, we suspect that the stabilization of IAA8 is achieved by unidentified post-translational modification (PTM). Various PTMs, such as phosphorylation, ubiquitination, and sumoylation, can alter protein stability, subcellular localization, and protein interactions in response to external signals (Yang et al., 2003). Interestingly, various Aux/IAA proteins, such as IAA1, IAA5, IAA8, IAA11, IAA13, IAA15, and IAA31, are putative substrates of kinases (Popescu et al., 2009). Therefore, there is a strong possibility that IAA8 is stabilized by cold and ROS through phosphorylation during seed germination.

### IAA8 Promotes Seed Germination Through Suppression of *ABI3* Transcription

*ABI3* is a well-known key negative regulator in seed germination (Liu et al., 2013). *ABI3* is transcriptionally induced by ABA and involved in ABA-mediated inhibition of seed germination (Bentsink and Koornneef, 2008; Liu et al., 2013). Auxin also negatively regulates seed germination through auxin response factor 10 (ARF10)- and ARF16-mediated increase of *ABI3* transcription (Nemhauser et al., 2006; Santner and Estelle, 2009; Liu et al., 2013). We showed that the *iaa8-1* mutant has delayed seed germination and higher levels of *ABI3*, *ABI4*, *ABI5*, *Em1*, and *Em6* transcript (**Figures 4A–E**), while the *RAV1* transcript level was markedly repressed in *iaa8-1* mutant compared to wild type (**Figure 4F**). Similar observations in seeds of various auxin biosynthesis and signaling mutants (Liu et al., 2013) demonstrate that auxin signaling can negatively control seed germination through ARF-mediated regulation of *ABI3* transcription. In addition, exogenous auxin application up-regulates the transcription of *ABI3*, resulting in inhibition of seed germination (Liu et al., 2007; Liu et al., 2013). By contrast, the *arf10arf16* double mutant shows enhanced germination with reduced transcription levels of *ABI3* (Liu et al., 2013), suggesting that ARF10 and ARF16 are required for maintenance of *ABI3* transcription. Moreover, ABI3 transcription factor positively regulates *ABI4* and *ABI5* expression, which in turn promotes *Em1* and *Em6* transcription, thereby, negatively regulating seed germination (Soderman et al., 2000; Skubacz et al., 2016). In contrast, the higher transcript level of *RAV1* positively regulates seed germination (Feng et al., 2014). Taken together, these results suggest that IAA8 acts as a key upstream regulator during seed germination. Additionally, ChIP assay confirmed that IAA8 associates to *ABI3* promoter, specifically to AuxRE motif *via* unidentified interacting ARFs (**Figure 5**). These observations indicate that *ABI3* transcription can be down-regulated through IAA8-mediated transcriptional inactivation of ARFs proteins. Interestingly, various ARFs, such as ARF4, ARF5, ARF6, ARF7, ARF8, ARF11, ARF14, ARF15, ARF16, ARF19, ARF20, and ARF22 have been previously investigated as IAA8 interacting partners (Vernoux et al., 2011; Arase et al., 2012; Wang et al., 2013; Piya et al., 2014). Therefore, we suspect that stabilized IAA8 down-regulates transcription of *ABI3* by inactivating ARF proteins, which directly bind to the promoter of *ABI3*. A better understanding of auxin signaling in seed germination will be achieved by investigating which specific ARF proteins bind to IAA8 and regulate the transcription of *ABI3*.

**Figure 6:**
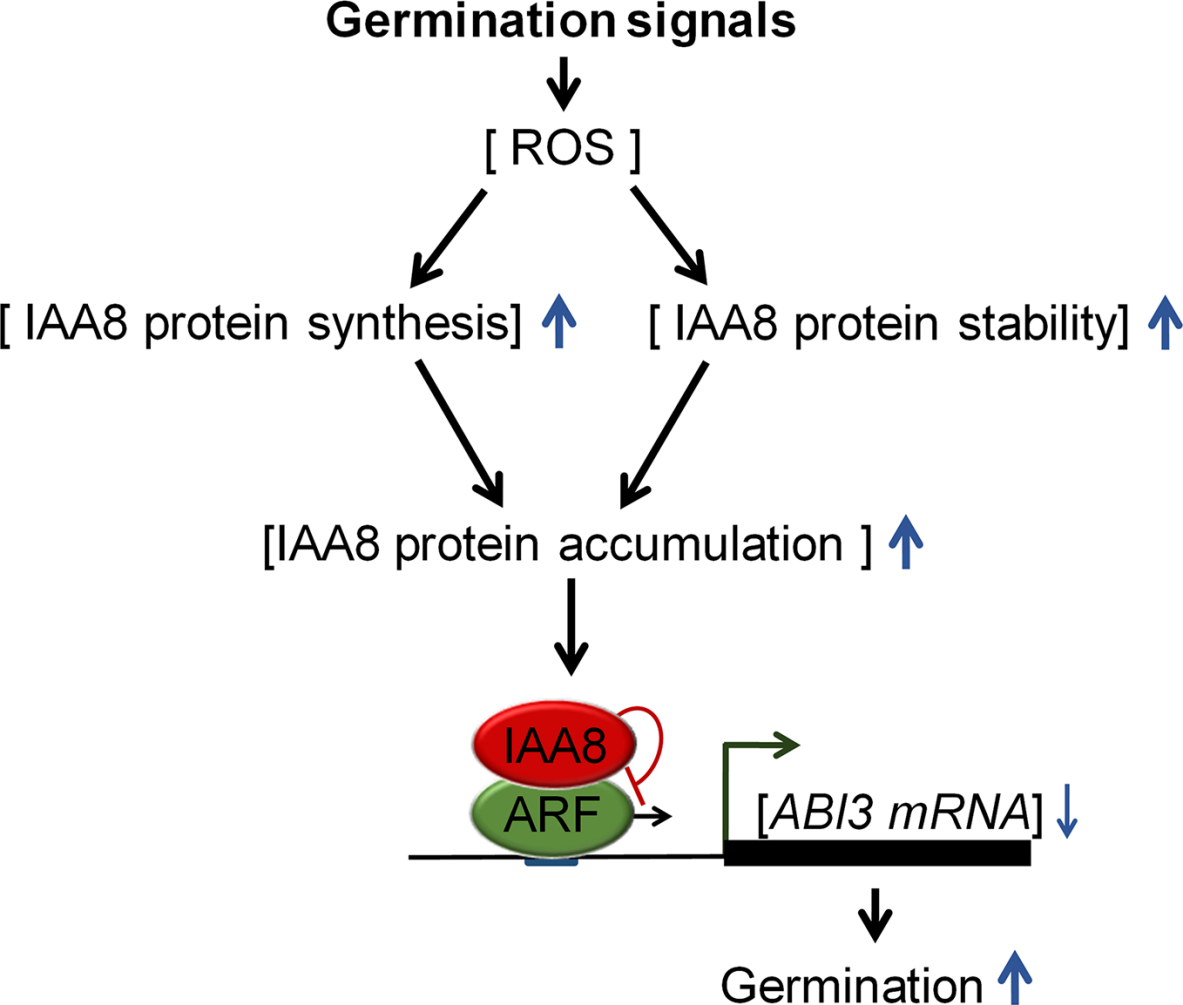
Proposed model explaining the role of IAA8 in seed germination. In response to germination signals, ROS accumulate in seeds. *IAA8* gene expression is induced in response to these signals, thereby enhancing IAA8 protein synthesis. In addition, the IAA8 protein is stabilized by cold- and ROS-mediated signaling pathways, triggering the accumulation of IAA8 protein. The accumulated IAA8 inhibits ARF activity, thereby down-regulating *ABI3* gene expression, which ultimately promotes seed germination. Upward and downward blue arrows represent enhanced and reduced levels, respectively.

We propose a model (**Figure 6**) explaining how Aux/IAA proteins regulate seed germination. Germination-inducing signals promote seed germination by increasing ROS, which triggers ABA catabolism and GA biosynthesis (Liu et al., 2010). In response to these signals, IAA8 accumulates not only by induction of protein synthesis, but also by protein stabilization. The accumulated IAA8 increases seed germination through transcriptional inhibition of *ABI3* by inactivating ARFs activity.

## Data Availability Statement

The raw data supporting the conclusions of this article will be made available by the authors, without undue reservation, to any qualified researcher.

## Author Contributions

SH, SK, and WC conceived the study. XN and WC supervised this study. SH and SB performed the experiments. SH, SK, and AA analyzed the data. SH, AA, DJ-Y, and WC wrote the manuscript with feedback from all authors.

## Funding

This work was supported by the Next-Generation BioGreen 21 Program (#PJ01325401) funded by RDA and the National Research Foundation of Korea (NRF) grant funded by the Korean government (MSIP) (no. 2019R1A2C1009932), partly by the Basic Science Research Program through the National Research Foundation of Korea (NRF) funded by the Ministry of Education (no. 2018R1A6A3A11042628), and by the Vietnam National Foundation for Science and Technology Development (NAFOSTED) under grant number 106.02-2017.09. SH and SB were supported by the Brain Korea 21 Plus (BK21+) fellowship program.

## Conflict of Interest

The authors declare that the research was conducted in the absence of any commercial or financial relationships that could be construed as a potential conflict of interest.

## Abbreviations

ABA, Abscisic Acid; *ABI3*, *ABSCISIC ACID INSENSITIVE3;* ARF, Auxin Response Factor; Aux/IAA, Auxin/Indole-3-Acetic Acid; GA, Gibberellic Acid; ROS, Reactive Oxygen Species.
