# Supplementary material for: The Auxin Signaling Repressor IAA8 Promotes Seed Germination Through Down-Regulation of ABI3 Transcription in Arabidopsis
Source: Front Plant Sci. 2020 Feb 20;11:111. doi: 10.3389/fpls.2020.00111 (PMC7045070; doi:10.3389/fpls.2020.00111)
Supplement: Supplementary file 1 [file Presentation_1.pdf]

Supplementary Figures

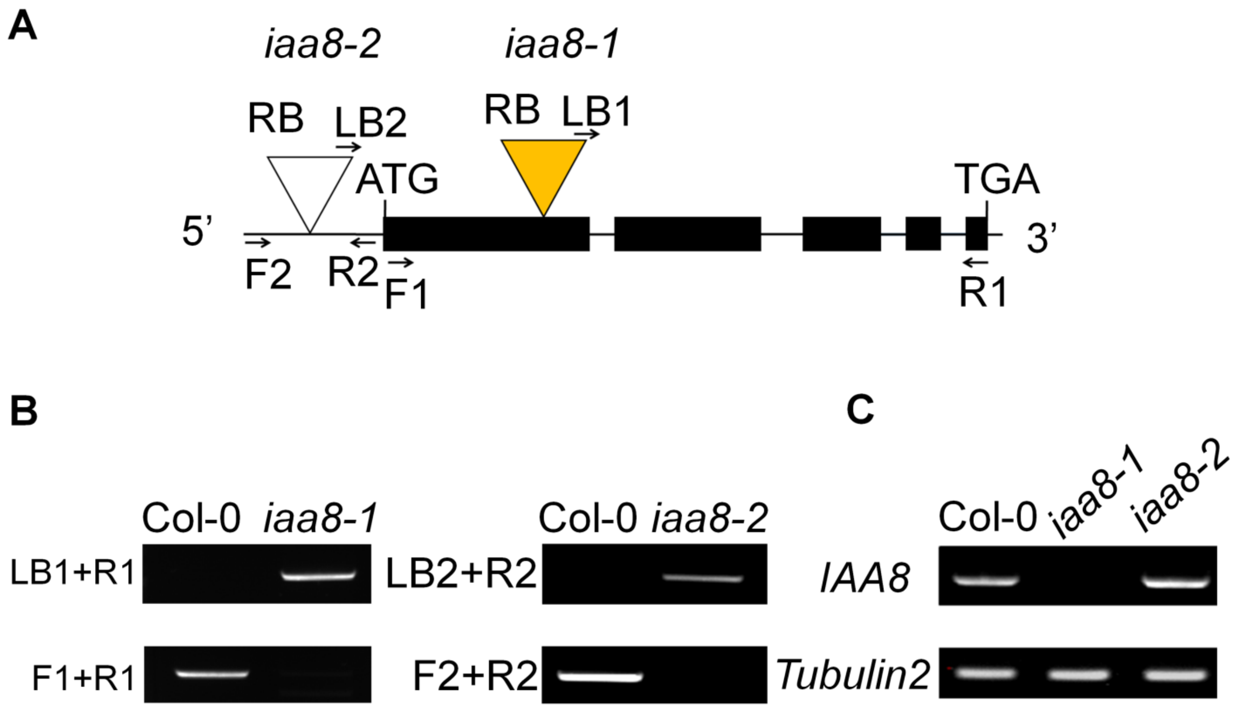

Supplementary Figure 1 Isolation and genotyping of T-DNA insertion mutants. **(A)** Schematic representation of *IAA8* gene structure. Boxes represent exons, and solid lines represent introns. The T-DNA insertion is shown as a yellow triangle (*iaa8-1*) or white triangle (*iaa8-2*). F1 and R1 are gene-specific forward and reverse primers, respectively, while F2 and R2 are forward and reverse primers in the region upstream of ATG. LB, primer specific to the T-DNA left border. **(B)** Genotyping analysis of T-DNA insertion mutant lines *iaa8-1* and *iaa8-2*. Primers used for PCR were those shown in **(A)**. **(C)** semi-quantitative RT-PCR analysis of the *IAA8* transcript in Col-0, *iaa8-1*, and *iaa8-2* mutant seedlings. Total RNA was extracted from 10-day-old seedlings and semi-quantitative RT-PCR analysis was performed using gene-specific primers; *Tubulin2* was used as an internal control.

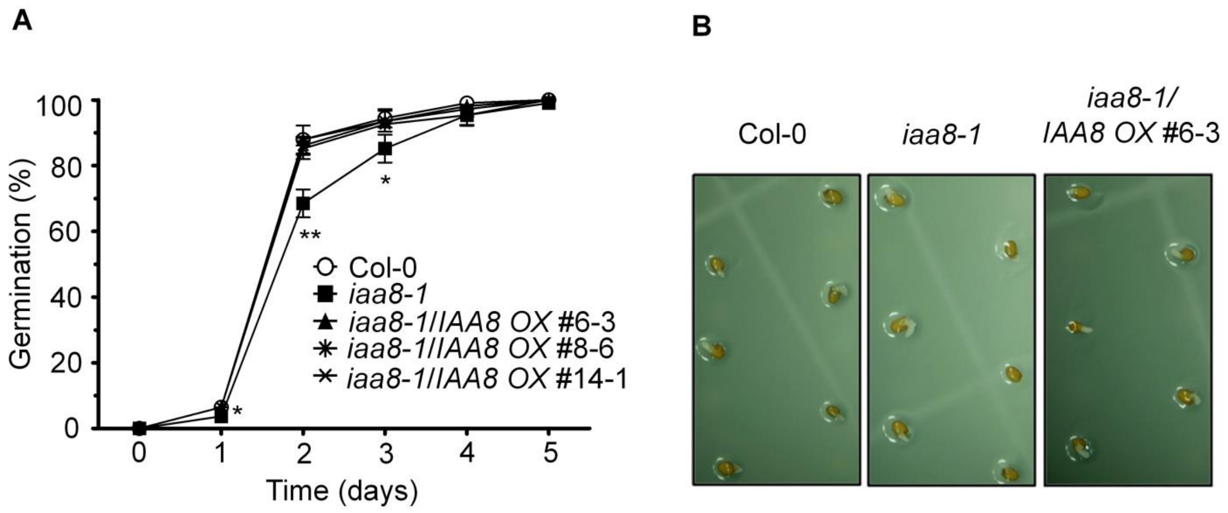

Supplementary Figure 2 Germination of Col-0, *iaa8-1*, and *iaa8-1*/*IAA8 OX* complemented seeds. Seeds of the selected genotype were allowed to germinate on ½ MS medium. **(A)** Radicle protrusion was monitored at the indicated times (days). Data are presented as mean values. Error bars represent SE. Significant difference was determined by Student's t-test (*P < 0.05 and **P < 0.01). **(B)** Representative images of seeds taken after 3 days of growth on ½ MS medium.

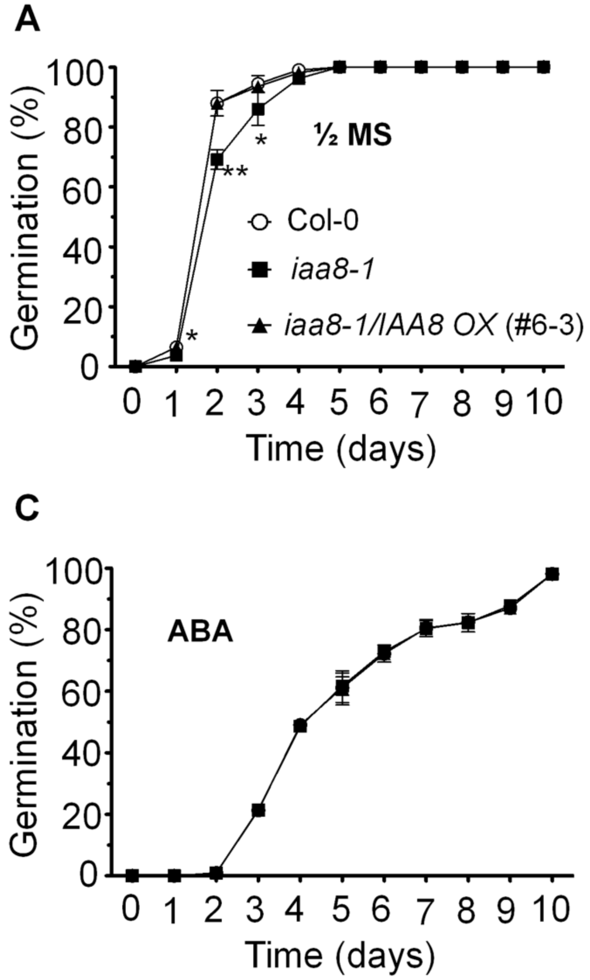

Supplementary Figure 3 Effect of auxin on seed germination of Col-0, *iaa8-1* and *iaa8-1/IAA8 OX* (#6-3) in presence and absence of ABA. Seeds of the selected genotype were stratified and allowed to germinate on **(A)** ½ MS medium or ½ MS supplemented with **(B)** 5 μM NAA. **(C)** 1 μM ABA and **(D)** 5 μM NAA and 1 μM ABA together for the indicated time period (days). Radicle protrusion was monitored at the indicated times (days). Data are presented as mean values. Error bars represent SE. Significant difference was determined by Student’s t-test (*P < 0.05 and **P < 0.01).
