# Supplementary material for: The Auxin Signaling Repressor IAA8 Promotes Seed Germination Through Down-Regulation of ABI3 Transcription in Arabidopsis
Source: Front Plant Sci. 2020 Feb 20;11:111. doi: 10.3389/fpls.2020.00111 (PMC7045070; doi:10.3389/fpls.2020.00111)
Supplement: Supplementary file 2 [file Table_1.docx]

**Supplementary Tables**

**Supplementary Table 1** List of primers used for genotyping.

| **Name** | **Position** | **Sequence** |
| --- | --- | --- |
| *IAA8* | F1 | 5'- *ATGTCTTATCGATTGCTAAG* -3' |
|  | R1 | 5'- *TCAAACCCGCTCTTTGTT* -3' |
|  | F2 | 5'- *ACCGATACGTGGTCCCATAAC* -3' |
|  | R2 | 5'- *GAACCAAACAAACAAACCCAG* -3' |
| T-DNA | LB | *CCAGCGTGGACCGCTTGCTGCAACTCTCTC* |
