# Supplementary material for: The Auxin Signaling Repressor IAA8 Promotes Seed Germination Through Down-Regulation of ABI3 Transcription in Arabidopsis
Source: Front Plant Sci. 2020 Feb 20;11:111. doi: 10.3389/fpls.2020.00111 (PMC7045070; doi:10.3389/fpls.2020.00111)
Supplement: Supplementary file 3 [file Table_2.docx]

**Supplementary Table 2** Primers used for RT-qPCR, semi-quantitative RT-PCR and ChIP-PCR.

| **Name** | **Position** | **Sequence** |
| --- | --- | --- |
| *IAA8* | F | 5'- *GGTCAATGTGGTCTTCATGG* -3' |
|  | R | 5'- *ACCTGGAGCTAAGCCAATAG* -3' |
| *ABI3* | F | 5'- *TTTGGAACATGCGCTACAGG* -3' |
|  | R | 5'- *TTGTCTCTTCGTAGCTGCTG* -3' |
| *ABI4* | F | 5'- *GGGCAGGAACAAGGAGGAAGTG* -3' |
|  | R | 5'- *ACGGCGGTGGATGAGTTATTGAT* -3' |
| *ABI5* | F | 5'- *ATGATCAAGAACCGCGAGTCTGC* -3' |
|  | R | 5'- *CGGTTGTGCCCTTGACTTCAAAC* -3' |
| *Em1* | F | 5'- *GGGACGTAAAGGAGGACTCAGTA* -3' |
|  | R | 5'- *CTTTGACTCATCGATCTCAATCC* -3' |
| *Em6* | F | 5'- *GGAGCAGTTAGGAACTGAAGGAT* -3' |
|  | R | 5'- *TTTGGATTCGTCTATCTCGACTC* -3' |
| *RAV1* | F | 5'- *TCCGGGTCAGATTTAGATGC* -3' |
|  | R | 5'- *AAGATGCGTTGCTTCTTGCT* -3' |
| *Tubulin2* | F | 5'- *CCAACAACGTGAAATCGACAG* -3' |
|  | R | 5'- *TCTTGGTATTGCTGGTACTCT* -3' |
| *ABI3*-P1 | F | 5'- *CAAGGTCGAACCCTGTTTGT* -3' |
|  | R | 5'- *AAGTGGAAGAGGCAGACACG* -3' |
| *ABI3*-P2 | F | 5'- *TGTTTGTAAGTATTATGAGCT* -3' |
|  | R | 5'- *AGTTATTCTCTTTCTCTTTGC* -3' |
